# Depression treatment decreases healthcare expenditures among working age patients with comorbid conditions and type 2 diabetes mellitus along with newly-diagnosed depression

**DOI:** 10.1186/s12888-016-0964-9

**Published:** 2016-07-19

**Authors:** Rituparna Bhattacharya, Chan Shen, Amy B. Wachholtz, Nilanjana Dwibedi, Usha Sambamoorthi

**Affiliations:** Humana Inc, Clinical Data Analytics (CODA), Tucson, AZ USA; Department of Health Services Research and Biostatistics, University of Texas MD Anderson Cancer Center, Houston, TX USA; Department of Psychiatry, University of Massachusetts Medical School, Worcester, MA USA; Department of Pharmaceutical Systems and Policy, School of Pharmacy, West Virginia University, Morgantown, WV USA; Department of Social & Behavioral Sciences, School of Public Health, West Virginia University, Morgantown, WV USA; Departments of Health Services Research, The University of Texas MD Anderson Cancer Center, 1400 Pressler Street, Unit 1444, Houston, TX 77030 USA

**Keywords:** Depression, Type 2 diabetes mellitus, Healthcare expenditures, Comorbidity

## Abstract

**Background:**

There are many studies in the literature on the association between depression treatment and health expenditures. However, there is a knowledge gap in examining this relationship taking into account coexisting chronic conditions among patients with diabetes. We aim to analyze the association between depression treatment and healthcare expenditures among adults with Type 2 Diabetes Mellitus (T2DM) and newly-diagnosed depression, with consideration of coexisting chronic physical conditions.

**Methods:**

We used multi-state Medicaid data (2000–2008) and adopted a retrospective longitudinal cohort design. Medical conditions were identified using diagnosis codes (ICD-9-CM and CPT systems). Healthcare expenditures were aggregated for each month for 12 months. Types of coexisting chronic physical conditions were hierarchically grouped into: dominant, concordant, discordant, and both concordant and discordant. Depression treatment categories were as follows: antidepressants or psychotherapy, both antidepressants and psychotherapy, and no treatment. We used linear mixed-effects models on log-transformed expenditures (total and T2DM-related) to examine the relationship between depression treatment and health expenditures. The analyses were conducted on the overall study population and also on subgroups that had coexisting chronic physical conditions.

**Results:**

Total healthcare expenditures were reduced by treatment with antidepressants (16 % reduction), psychotherapy (22 %), and both therapy types in combination (28 %) compared to no depression treatment. Treatment with both antidepressants and psychotherapy was associated with reductions in total healthcare expenditures among all groups that had a coexisting chronic physical condition.

**Conclusions:**

Among adults with T2DM and chronic conditions, treatment with both antidepressants and psychotherapy may result in economic benefits.

## Background

It is well appreciated that depression can increase the medical expenditure of patients with chronic physical conditions such as type 2 diabetes mellitus or cardiovascular disease. Indeed, individuals with coexisting type 2 diabetes mellitus (T2DM) and depression use more healthcare services including inpatient care [12], outpatient care [11, 30], and prescription drug use [11, 30]. These patients’ records also show higher total medical expenses [6, 11, 12], as well as T2DM-related medical care expenditures, when compared to individuals with T2DM and no depression [23]. Indeed, the coexistence of depression and diabetes in patients is associated with 4.5-fold higher healthcare expenditures compared to patients without depression [11]. However, successful depression treatment has been associated with lower subsequent healthcare utilization and expenditures [40].

Randomized clinical trials have studied whether depression treatment delivered in primary care-based collaborative care settings among individuals with both depression and T2DM reduces healthcare expenditures when compared to usual care, in which referrals to outside mental healthcare professionals are given [19, 22, 39]. In collaborative healthcare settings, both depression and T2DM are managed with the help of coordinated healthcare teams comprised of primary care physicians, nurses, and other specialists. For example, the Pathways Study **[**22, 39], which included participants with diabetes and coexisting depression, found lower total healthcare expenditures at the end of 2-year and 5-year periods in the intervention group that received a 12 month stepped-care depression management program when compared to the control group that received usual care. In addition, a few observational studies have examined the association between depression treatment and healthcare expenditures among individuals with other chronic conditions. Using administrative claims data, one study showed that among individuals with dyslipidemia, T2DM, and coronary artery disease, existing either alone or as comorbid conditions, antidepressant medication adherence improved adherence to coexisting disease medications and thus reduced one-year healthcare expenditures [18]. In contrast, a study using elderly Medicare patient data found that beneficiaries with coexisting depression and chronic disease who received depression treatment had higher expenditures compared to patients not receiving depression treatment [5].

To summarize findings so far, randomized controlled trials have demonstrated that depression treatment delivered in collaborative care settings reduces expenditures among individuals with T2DM and coexisting depression, while studies using real world observational data have reported inconsistent findings. However, these studies were not specific to T2DM patients and also included special patient populations such as the elderly. It remains to be established whether depression treatment with antidepressants and psychotherapy, either alone or in combination, reduces healthcare expenditures among individuals with T2DM. Therefore, the primary objective of this study was to examine the association between the depression treatment method and healthcare expenditures among working age Medicaid beneficiaries with T2DM and newly-diagnosed depression. Additionally, to the best of our knowledge the literature is void on whether this association varies by coexisting chronic physical conditions. With the majority of adults (88.6 %) with T2DM in the U.S. having at least one additional chronic condition, and 15 % having reported four or more comorbid chronic conditions [7], the presence of comorbid chronic physical conditions among individuals with T2DM is a norm rather than an exception. Therefore, it is important to examine whether the relationship between depression treatment and expenditures varies by the type of coexisting chronic physical condition. It is particularly important to study this association in the working age population given that, in recent years, the prevalence of multiple coexisting chronic conditions has been increasing among working age adults [29, 43]. The primary objective of the study is examine whether the associations between depression treatment and T2DM-related, as well as total, healthcare expenditures vary by the type of coexisting chronic physical condition among working age Medicaid beneficiaries with T2DM and newly-diagnosed depression.

## Methods

### Study design

A retrospective longitudinal study with repeated measures design was used.

### Data source

#### Medicaid Analytic Extract (MAX) files

The MAX files are prepared and produced by the Centers for Medicare and Medicaid Services. Person-level data such as eligibility, demographics, managed care enrollment, a utilization summary, and Medicaid payments for enrollees are provided in the enrollment (“personal summary”) file. Information on International Classification of Diseases 9^th^ revision (ICD-9-CM codes) of conditions diagnosed, healthcare service utilizations, and charges paid by Medicaid for the services can be extracted from inpatient and other therapy files. Information on prescription drug use is provided in the pharmacy file. This study used Medicaid data from 2000–2008 from three states: New York (NY), Texas (TX), and Illinois (IL). These states were chosen to capture the diverse geographic and racial/ethnic populations represented by Medicaid. These states were among the largest state in terms of the number of Medicaid enrollees and include very diverse geographic and racial/ethnic populations. There have been many studies using Medicaid data from a few states [35, 49].

#### Area Health Resource Files (AHRF)

The AHRF contains national county-level health resource information on more than 6000 county level variables and is provided by the U.S. Department of Health and Human Services (2011) [48]. The Federal Information Processing Standard county codes, which are available both in the AHRF file and the personal summary file of the MAX data, were used to link the two files.

### Identification of the T2DM and newly-diagnosed depression study cohort

Seven longitudinal cohorts were identified: 2000–02, 2001–03, 2002–04, 2003–05, 2004–06, 2005–07, 2006–08. The study sample was comprised of working age adults aged 18–64 years with type 2 diabetes mellitus and at least one coexisting dominant, concordant, or discordant chronic physical condition and who were alive, not dually eligible for Medicare, and continuously enrolled in fee-for-service Medicaid for at least 24 months (*N* = 5295). Adults in the age group 18 and 64 years are defined as working age individuals. We describe our cohort creation in detail below.

#### Medicaid beneficiaries with T2DM

Medicaid enrollees with at least one inpatient visit or two or more physician outpatient visits which were at least 30 days apart, with a primary or secondary diagnosis of ICD-9-CM codes 250.x0 or 250.x2 during a calendar year, were identified as having T2DM. Medicaid beneficiaries with T2DM who had a diagnosis of depression or antidepressant medication use during the calendar year of their T2DM diagnosis as per our identification procedure were excluded.

#### Medicaid beneficiaries with newly-diagnosed depression

The eligible study population was followed into the subsequent calendar year to identify cases of newly-diagnosed depression. Enrollees with at least one outpatient physician visit or an inpatient admission with a primary or secondary diagnosis of depression in the following calendar year were classified as having newly-diagnosed depression [42]. Depression was identified using ICD-9-CM codes: 296.2 (major depressive disorder, single episode), 296.3 (major depressive disorder, recurrent episode), 311 (depression not elsewhere classified), 309.1 (prolonged depressive reaction), 300.4 (neurotic depression) and 298.0 (depressive type psychosis). These ICD-9-CM codes are extensively used by health plans to identify depression and have also been used in previous studies on depression in Medicaid enrollees [36, 37, 44]. Those with no newly-diagnosed depression were excluded from the study cohort. The first observed date of outpatient visit or inpatient discharge with a diagnosis of depression was the “index date”; enrollees had to be free of a depression diagnosis or antidepressant medication prescription 365 days prior to the index date. Although other studies have used 120 day depression-free periods to define newly-diagnosed depression [46], our 365 day look-back period was used with the intent to minimize misclassification of an episodic manifestation of chronic depression (where depression symptoms last for two or more years) as newly-diagnosed depression.

Additional exclusion criteria were: (1) not having a diagnosis of at least one chronic physical condition (identified by ICD-9-CM codes included in Appendix) in the baseline period; (2) no continuous fee-for-service Medicaid eligibility; (3) enrollment in Medicare at any point during the observation period; (4) dying during the study period; and (5) not using inpatient or outpatient Medicaid services during the study period. We excluded patients who did not use inpatient or outpatient Medicaid services because we cannot capture type of depression treatment, coexisting conditions, and healthcare expenditure for these individuals. We included only those enrolled in fee-for-service Medicaid program because healthcare experience of adults who are not enrolled in fee-for-service cannot be captured. We excluded patients who died during the study period as they have a shorter observation time and healthcare expenditures peak during the time period before death. Therefore, our estimates could be underestimated.

### Dependent variables

#### Total healthcare expenditures

Total healthcare expenditure per person included in the study cohort was defined as the total dollar amount that Medicaid paid for inpatient, outpatient, and pharmacy claims.

#### T2DM-related healthcare expenditures

T2DM-related healthcare expenditure per person included in the study cohort was defined as the total dollar amount that Medicaid paid for inpatient and outpatient claims with a diagnosis of T2DM. The T2DM-related healthcare expenditures were also identified on a monthly basis during the 12 month follow-up period.

The total and T2DM-related healthcare expenditures were adjusted by the medical component of the Consumer Price Index (CPI) and are expressed in 2008 constant dollars. After assessing the skewness and kurtosis properties and linearity through qq-plots, the expenditure variables were log transformed and used as dependent variables.

### Independent variables

The independent variables were chosen based on a behavioral model of factors that influence the use of health services known as the Anderson Behavioral Model (ABM) [2]. The ABM posits that utilization of health services varies as a function of (1) each individual’s unique predisposition for using services (predisposing factors); (2) the means available to each individual for obtaining services (enabling factors); (3) each individual’s level of need; (4) personal health practices; and (5) the external environment. Based on the ABM, we used a breadth of independent variables in our study. Here we provide detailed information on several variables associated with need, followed by additional independent variables congruent with the ABM.

#### Key independent variables: depression treatment during the acute phase (need factor)

The first four months following newly-diagnosed depression is known as the acute phase of depression treatment [47]. The initial treatment choice may influence the effectiveness of depression treatment and therefore may also be associated with healthcare expenditures over time. Single-modality depression treatment with either antidepressants or psychotherapy could be used during the acute phase, as could combination treatment.

#### Antidepressant use

The national drug codes available in the prescription drug use files of MAX data were used to identify the different classes of antidepressant drugs: selective serotonin reuptake inhibitors, selective norepinephrine reuptake inhibitors, tricyclic antidepressants, monoamine oxidase inhibitors and other (mirtazapine and bupropion). *Psychotherapy Use:* The use of psychotherapy was identified with Current Procedural Terminology (CPT) codes. The following psychotherapy types were used: (1) psychotherapy diagnostic interview (90801, 90802); (2) individual psychotherapy [individual psychotherapy 20–30 min (90804, 90816, 90805, 90817), 45–50 min (90806, 90818, 90807, 90819), 75–80 min (90808, 90821, 90809, 90822), interactive individual psychotherapy 20–30 min (90810, 90823, 90811, 90824), 45–50 min (90812, 90826, 90813, 90827), 75–80 min (90814, 90828, 90815, 90829)]; (3) other psychotherapy [family psychotherapy (90846, 90847, 90849), group psychotherapy (90853), interactive group psychotherapy (90857)] [16].

Depression treatment in the acute phase was categorized as treatment with: (1) antidepressants: these individuals received at least one prescription for antidepressants, but no psychotherapy visits; (2) psychotherapy: those who received at least one psychotherapy office visit but no prescription for antidepressant drugs; (3) both antidepressants and psychotherapy: those who received a minimum of one prescription for antidepressants and one psychotherapy visit; and (4) no treatment: these individuals did not receive a prescription for antidepressants or psychotherapy office visits.

#### Coexisting chronic physical condition types (need factor)

As other physical conditions coexisting with T2DM often impact the medical care, self-management, and healthcare outcomes of an individual with T2DM, Piette and Kerr [34] developed a framework that classified coexisting conditions among individuals with T2DM into categories based on similarities and differences from T2DM pathophysiology and management. The categories of conditions that might coexist with T2DM were defined as: dominant (conditions whose severity eclipses all other conditions’ management plans, such as metastatic cancer), concordant (conditions that overlap with T2DM in their pathophysiology and management plans such as cardiovascular diseases), or discordant (conditions with unrelated pathophysiology or management plans such as musculoskeletal disorders) [34].

Following this framework, it is likely that the presence of coexisting conditions among individuals with T2DM and newly-diagnosed depression may affect depression management and the response to depression treatment. Therefore, based on this theoretical framework, forty-four different coexisting chronic physical condition types (Appendix) were identified. A hierarchical classification was followed [18], and dominant conditions were given priority because they often take precedence over the management of other health conditions. Only among those without dominant conditions, concordant and discordant conditions were identified. The types of coexisting chronic physical conditions were classified as: 1) dominant conditions; 2) concordant; 3) discordant; and 4) both concordant and discordant.

#### Other independent variables

*Predisposing Factors:* The included variables were based on demographics (gender, age, race/ethnicity [Whites, African Americans, Hispanics or other races]). *Enabling Factors:* Using Medicaid eligibility status, the enabling factors included were: eligibility due to poverty (yes/no), medical needs (yes/no) and waiver (yes/no). *Need Factors:* These included other mental health conditions such as bipolar disorder, schizophrenia, post-traumatic stress disorder, and alcohol and drug abuse. *Personal Health Practices:* Healthcare-seeking behaviors were used as a measure for personal health practices and included baseline healthcare utilization characteristics such as number of emergency room visits during the 180 days prior to the index date, inpatient hospitalization, number of oral antidiabetic medication classes and insulin use as identified by NDC codes, presence of polypharmacy identified by use of six or more drug classes in the 90 days prior to the index date, number of outpatient visits measured in quartiles and total baseline healthcare expenditures. *External Environment:* External environment variables included state of residence, community level healthcare infrastructure (presence of a community mental health clinic (CMHC) and a federally qualified health clinic (FQHC) in a county, whether county of residence was designated as a Health Professional Shortage area (HPSA), and density of social workers in a county), and community level social determinants of health variables (urban/rural status of a county, median income in the county, indicators for whether the percentage below poverty level and percentage with college education in the county were different than national averages based on U.S. census estimates). *Other variables:* After depression treatment response is achieved during the acute phase, treatment for depression may be continued for another 4 to 9 months in the continuation phase. Therefore, it may be plausible that some individuals received depression treatment with antidepressants and/or psychotherapy during the entire length of follow-up, whereas some people had a shorter treatment period. To control for such variation, the statistical models additionally controlled for *Antidepressant treatment at each month of follow-up:* This was defined as a dichotomous (yes/no) variable, which indicated whether an individual received an antidepressant prescription during each month of the follow-up; *Psychotherapy treatment at each month of follow-up:* A categorical (yes/no) variable that indicated whether an individual received outpatient psychotherapy during each month of the follow-up; Y*ear of observation:* As data from multiple years forming seven different panels were used (2000–2002, 2001–2003, 2002–2004, 2003–2005, 2004–2006, 2005–2007, 2006–2008), a variable indicating which particular cohort the observation belonged to was also included.

### Statistical analyses

We provide descriptive statistics, including frequency, mean, standard errors, and chi-square statistics. As healthcare expenditures were aggregated for each month of follow-up, 12 observations were available for each individual. Due to repeated measures of healthcare expenditures, the observations were not independent. Because standard regression techniques assume that individual observations are independent, they cannot be applied to data with repeated measures. Therefore, the multivariable analyses consisted of linear mixed effects models which accounted for correlated error terms of observations from the same person. More specifically, we adjusted for both random effects (a random intercept) and fixed effects including time in months, depression Treatment, predisposing factors (gender, age, race/ethnicity), need factors (other mental health conditions, coexisting chronic physical condition type), enabling factors (Medicaid eligibility -poverty, medical need, waiver), personal health practices (number of ER and outpatient visits, inpatient hospitalization, number of OAD classes, insulin use and polypharmacy), external environment characteristics (whether county of residence had a CMHC, FQHC, was HPSA for mental health, density of social workers, rural/urban status of county, median income in the county, whether percent below poverty level and percent with college education were greater than the national average), and other variables (antidepressant treatment at each month of follow-up, psychotherapy treatment at each month of follow-up and year of observation). Separate linear mixed model regressions on log of expenditures for each coexisting chronic physical condition type were conducted to examine whether the association between depression treatment and total and T2DM-related healthcare expenditures varied by comorbid condition type.

#### Adjusting for observed selection bias

Depression treatment is a choice variable and characteristics of the study population can influence this choice. To account for such observed differences, the inverse probability of treatment weighting (IPTW) was used. We used a logistic regression model to generate the estimated probability of treatment (i.e. propensity). The IPTW gives weight to each individual based on the inverse of their propensity to use a particular type of depression treatment. This helps to balance the probability of treatment across the treatment groups. In order to account for group size differences of the treatment groups, the weights were further stabilized by dividing them with the sample size of each group.

All analyses were conducted using Statistical Analysis Software (SAS 9.3).

## Results

In the study population (*N* = 5295), 36.3 % were aged 45–54 years, 38.5 % were between 55–64 years of age, and 25.2 % were aged 18–44 years; 67.3 % were female and 32.7 % were male; 26.8 % were white, 30.1 % were African American, and 43 % belonged to another race; the majority (89.1 %) lived in metro areas. 16.8 % lived in counties that were designated as shortage areas for mental health professionals and 51.1 % lived in counties without a community mental health clinic. A description of the study population is presented in Table 1.

**Table 1:** Description of the study population by coexisting chronic physical condition type among medicaid beneficiaries with type 2 diabetes mellitus and newly diagnosed depression. Multi-state medicaid claims database – 2000–2008

		All		Dominant		Concordant only		Discordant only		Both concordant & discordant		Sig
		N	%	N	%	N	%	N	%	N	%	
All		5295		753	14.2	1444	27.3	834	15.8	2264	42.8	***
Predisposing factors												
Age												
	18–44	1335	25.2	122	9.1	426	31.9	256	19.2	531	39.8	
	45–54	1921	36.3	277	14.4	457	23.8	317	16.5	870	45.3	
	55–64	2039	38.5	354	17.4	561	27.5	261	12.8	863	42.3	
Sex												***
	Female	3565	67.3	437	12.3	949	26.6	613	17.2	1566	43.9	
	Male	1730	32.7	316	18.3	495	28.6	221	12.8	698	40.3	
Race												**
	White	1420	26.8	203	14.3	369	26.0	251	17.7	597	42.0	
	AA	1596	30.1	240	15.0	396	24.8	250	15.7	710	44.5	
	Other	2279	43.0	310	13.6	679	29.8	333	14.6	957	42.0	
Need factor												
Other mental health conditions												***
	Yes	1877	35.4	349	18.6	476	25.4	294	15.7	758	40.4	
	No	3418	64.6	404	11.8	968	28.3	540	15.8	1506	44.1	
Enabling factors												
Medicaid eligibility - poverty												**
	Yes	4620	87.3	1237	26.8	708	15.3	2014	43.6	661	14.3	
	No	675	12.7	207	30.7	126	18.7	250	37.0	92	13.6	
Medicaid eligibility - Medical Need												*
	Yes	638	12.0	181	28.4	108	16.9	238	37.3	111	17.4	
	No	4657	88.0	1263	27.1	726	15.6	2026	43.5	642	13.8	
Medicaid eligibility - Waiver												
	Yes	300	5.7	89	29.7	60	20.0	114	38.0	37	12.3	
	No	4995	94.3	1355	27.1	774	15.5	2150	43.0	716	14.3	
Personal health practices												
Oral Antidiabetic Drugs (OADs)												***
	1	1508	28.5	171	11.3	422	28.0	272	18.0	643	42.6	
	2	1238	23.4	144	11.6	353	28.5	214	17.3	527	42.6	
	3+	450	8.5	38	8.4	156	34.7	63	14.0	193	42.9	
	None	2099	39.6	400	19.1	513	24.4	285	13.6	901	42.9	
Insulin Use												***
	Yes	1827	34.5	277	15.2	512	28.0	148	8.1	890	48.7	
	No	3468	65.5	476	13.7	932	26.9	686	19.8	1374	39.6	
Polypharmacy												***
	Yes	1915	36.2	293	15.3	345	18.0	295	15.4	982	51.3	
	No	3380	63.8	460	13.6	1099	32.5	539	15.9	1282	37.9	
Inpatient hospitalization												***
	Yes	2604	49.2	534	20.5	532	20.4	232	8.9	1306	50.2	
	No	2691	50.8	219	8.1	912	33.9	602	22.4	958	35.6	
Outpatient visits												***
	1st quartile	1392	26.3	85	6.1	588	42.2	294	21.1	425	30.5	
	2nd quartile	1221	23.1	139	11.4	352	28.8	215	17.6	515	42.2	
	3rd quartile	1352	25.5	227	16.8	282	20.9	180	13.3	663	49.0	
	4th quartile	1330	25.1	302	22.7	222	16.7	145	10.9	661	49.7	
		Mean ± SE		Mean ± SE		Mean ± SE		Mean ± SE		Mean ± SE		
Number of T2DM-related office visits		6.93 ± 0.13		7.98 ± 0.29		6.51 ± 0.29		5.44 ± 0.21		7.40 ± 0.19		
Number of ER visits		1.18 ± 0.03		1.65 ± 0.11		0.66 ± 0.04		0.78 ± 0.05		1.51 ± 0.05		
External environment												
State												***
	Illinois	1502	28.4	211	14.0	379	25.2	256	17.0	656	43.7	
	New York	2550	48.2	402	15.8	725	28.4	405	15.9	1018	39.9	
	Texas	1243	23.5	140	11.3	340	27.4	173	13.9	590	47.5	
HPSA- mental health care												*
	Yes	4405	83.2	651	14.8	1209	27.4	679	15.4	1866	42.4	
	No	890	16.8	102	11.5	235	26.4	155	17.4	398	44.7	
Metro												**
	Yes	4719	89.1	698	14.8	1291	27.4	730	15.5	2000	42.4	
	No	576	10.9	55	9.5	153	26.6	104	18.1	264	45.8	
CMHC												
	Yes	2590	48.9	376	14.5	702	27.1	393	15.2	1119	43.2	
	No	2705	51.1	377	13.9	742	27.4	441	16.3	1145	42.3	
FQHC												**
	Yes	4365	82.4	651	14.9	1199	27.5	674	15.4	1841	42.2	
	No	930	17.6	102	11.0	245	26.3	160	17.2	423	45.5	
Median income												**
	1st quartile	1312	24.8	145	11.1	365	27.8	204	15.5	598	45.6	
	2nd quartile	1415	26.7	199	14.1	378	26.7	246	17.4	592	41.8	
	3rd quartile	1235	23.3	182	14.7	345	27.9	190	15.4	518	41.9	
	4th quartile	1333	25.2	227	17.0	356	26.7	194	14.6	556	41.7	
% with GT 4 years college education > 16%^a^												**
	Yes	4399	83.1	656	14.9	1197	27.2	704	16.0	1842	41.9	
	No	896	16.9	97	10.8	247	27.6	130	14.5	422	47.1	
% below poverty level GT 11.1%^b^				753		1444		834		2264		
	Yes	4692	88.6	675	14.4	1284	27.4	732	15.6	2001	42.6	
	No	603	11.4	78	12.9	160	26.5	102	16.9	263	43.6	
		Mean ± SE		Mean ± SE		Mean ± SE		Mean ± SE		Mean ± SE		
Density of Social Workers		3.01 ± 0.02		3.27 ± 0.06		3.02 ± 0.04		3.02 ± 0.06		2.91 ± 0.04		

Note: Study sample was comprised of adults with type 2 diabetes mellitus aged 18–64 years with at least one coexisting dominant, concordant, or discordant chronic physical condition and who were alive, not dually eligible for Medicare, and continuously enrolled in fee-for-service Medicaid for at least 24 months (*N* = 5295); includes Medicaid data from three states: Illinois, Texas, New York

Asterisks (*) represent significant differences in study population characteristics and coexisting chronic physical condition categories (e.g. Dominant, Concordant Only, Discordant Only, and Both Concordant and Discordant), derived from chi-square statistics

***P < .001; **.001 ≤ *P* < .01; *.01 ≤ *P* < .05

^a^16 % cutoff was chosen based on 2000 Census Education attainment results

^b^11.1 % cutoff was chosen based on 1999 Census Poverty in people aged 18–64 years results

*HPSA* Health Professional Shortage Area, *CMHC* Community Mental Health Clinic, *FQHC* Federally Qualified Health Clinic, *GT* Greater Than

In the study population, 14.2 % had a coexisting chronic dominant condition, 27.3 % had a concordant condition, 15.8 % had a discordant condition, and 42.8 % had both a concordant and discordant condition (see Methods for condition definitions). All individual baseline characteristics and the majority of the county level characteristics differed significantly among the comorbid chronic condition type. For example, a greater proportion of individuals with dominant conditions were older (17.4 % were aged 55–64 years vs 9.1 % in the 18–44 year age group), male (18.3 % vs 12.3 % female), had other mental health conditions in addition to newly-diagnosed depression (18.6 % vs 11.6 % with no other mental health condition), inpatient hospitalization (20.5 % vs 8.1 % for those with no inpatient care), and had a higher number of outpatient visits (22.7 % in the 4th vs 6.1 % in the 1st quartile). Table 1 also presents a description of the study population by type of coexisting chronic physical condition and contains additional features including Medicaid eligibility status and median income.

During the acute phase, 27.3 % of the study population had treatment with antidepressants, 18.1 % had treatment with psychotherapy, 11.4 % had treatment with both antidepressants and psychotherapy, and 43.2 % had no depression treatment. Unadjusted chi-square analyses revealed that depression treatment during the acute phase varied significantly (*P*-value <0.001) among the coexisting chronic physical condition subgroups. For example, treatment with both antidepressants and psychotherapy was received by 12.6 % of the study population with dominant conditions, 11.2 % with concordant conditions, 10.7 % with discordant conditions, and 11.4 % with both concordant and discordant conditions. These data are not presented in tabular form.

The mean total and T2DM-related healthcare expenditures for the 12 month period after depression diagnosis were $30,590 and $13,642, respectively, for the group given antidepressants, $35,099 and $15,654 for those treated with psychotherapy, $33,032 and $15,726 for the group treated with both antidepressants and psychotherapy, and $34,041 and $14,801 for those receiving no depression treatment. The mean monthly total and T2DM-related expenditures across all depression treatment categories decreased over time in both the overall population as well as within each coexisting chronic physical condition subgroup.

Table 2 presents the results of linear mixed model regression analyses which revealed that, compared to no depression treatment, all other treatment types were associated with a reduction in total healthcare expenditures. When compared to no depression treatment, depression treatment with only antidepressants was associated with 16 % (95 % CI: 10 %–23 %) reduction in total healthcare expenditures, treatment with only psychotherapy was associated with 22 % (95 % CI: 14 %–29 %) reduction in total healthcare expenditures and treatment with both antidepressants and psychotherapy was associated with 28 % (95 % CI: 19 %–36 %) reductions in total healthcare expenditures. Treatment with psychotherapy and both antidepressants and psychotherapy was associated with 28 and 18 % reduction in T2DM-related expenditures as compared to no depression treatment.

**Table 2:** IPTW adjusted association between depression treatment and healthcare expenditures, among medicaid beneficiaries with type 2 diabetes mellitus and newly-diagnosed depression multi-state medicaid claims database – 2000 – 2008

	ALL expenditures				T2DM-related expenditures			
Depression treatment	Change	95 % CI		Sig	Change	95 % CI		Sig
Only antidepressants	−0.16	−0.23	−0.10	***	−0.10	−0.18	0.00	
Only psychotherapy	−0.22	−0.29	−0.14	***	−0.28	−0.36	−0.19	***
Antidepressants and psychotherapy	−0.28	−0.36	−0.19	***	−0.18	−0.29	−0.06	**

Reference Group: No Depression Treatment

Note: Study sample was comprised of adults with type 2 diabetes mellitus aged 18–64 years with at least one coexisting dominant, concordant, or discordant chronic physical condition and who were alive, not dually eligible for Medicare, and continuously enrolled in fee-for-service Medicaid for at least 24 months (*N* = 5295); includes Medicaid data from three states: Illinois, Texas, New York

All healthcare expenditures included inpatient, outpatient, and prescription drug-related expenditures; T2DM- related expenditures included inpatient and outpatient expenditures due to T2DM-related diagnosis. The expenditures were log transformed

Asterisks indicate statistical significance and are based on mixed effects models; no antidepressant treatment is the reference group for dependent variable. ***P < .001; **.001 ≤ *P* < .01; *.01 ≤ *P* < .05

*T2DM* Type 2 Diabetes Mellitus; depression: Major Depressive Disorder, *IPTW* Inverse Probability Treatment Weights, *SE* Standard Error

The IPTW-adjusted association between depression treatment categories during the acute phase and healthcare expenditures varied by coexisting chronic physical condition type (Table 3). Treatment with only psychotherapy was associated with significant reductions in total healthcare expenditures and T2DM related Expenditures among those with dominant conditions and among those with both concordant and discordant conditions. Treatment with both antidepressants and psychotherapy was associated with reductions in total healthcare expenditures among all types of coexisting chronic physical condition groups.

**Table 3:** IPTW adjusted association between depression treatment and healthcare expenditures stratified by coexisting condition type. Among medicaid beneficiaries with type 2 diabetes mellitus and newly diagnosed depression. Multi-state medicaid claims database – 2000–2008

	ALL expenditures				T2DM-related expenditures			
Depression treatment categories	Change	95 % CI		Sig	Change	95 % CI		Sig
Dominant								
Only antidepressants	−0.23	−0.41	0.01		−0.23	−0.44	0.06	
Only psychotherapy	−0.48	−0.59	−0.34	***	−0.41	−0.55	−0.23	***
Antidepressants and Psychotherapy	−0.41	−0.57	−0.19	**	−0.24	−0.48	0.1	
Concordant ONLY								
Only antidepressants	−0.21	−0.33	−0.08	**	−0.05	−0.22	0.16	
Only psychotherapy	−0.11	−0.26	0.06		−0.19	−0.36	0.03	
Antidepressants and Psychotherapy	−0.23	−0.38	−0.04	*	−0.27	−0.44	−0.06	*
Discordant only								
Only antidepressants	−0.18	−0.31	−0.02	*	−0.09	−0.26	0.13	
Only psychotherapy	−0.09	−0.29	0.18		−0.1	−0.33	0.2	
Antidepressants and Psychotherapy	−0.29	−0.45	−0.08	*	−0.1	−0.35	0.23	
Both concordant & discordant								
Only antidepressants	−0.1	−0.2	0.01		−0.1	−0.23	0.06	
Only psychotherapy	−0.21	−0.31	−0.09	***	−0.34	−0.45	−0.19	***
Antidepressants and Psychotherapy	−0.27	−0.38	−0.15	***	−0.1	−0.28	0.14	

Reference Group: No Depression Treatment

Note: Study sample was comprised of adults with type 2 diabetes mellitus aged 18–64 years with at least one coexisting dominant, concordant, or discordant chronic physical condition and who were alive, not dually eligible for Medicare, and continuously enrolled in fee-for-service Medicaid for at least 24 months (*N* = 5295); includes Medicaid data from three states: Illinois, Texas, New York

All healthcare expenditures included inpatient, outpatient and prescription drug-related expenditures; T2DM-related expenditures included inpatient and outpatient expenditures due to T2DM-related diagnosis. The expenditures were log transformed

Asterisks indicate statistical significance and are based on mixed effects models; ***P < .001; **.001 ≤ *P* < .01; *.01 ≤ *P* < .05

*T2DM* Type 2 Diabetes Mellitus; depression: Major Depressive Disorder; *IPTW* Inverse Probability Treatment Weights, *SE* Standard Error

## Discussion

The study findings indicate that depression treatment is associated with reductions in total healthcare expenditures as compared to no depression treatment. Randomized clinical trials examining the effectiveness of depression treatment with antidepressants and/or psychotherapy in the T2DM patient population with coexisting depression have found that antidepressants reduce the symptoms of depression [1, 15, 24–26, 28, 32, 33]. Moreover, psychotherapy (e.g. cognitive behavioral therapy) has also been shown to be effective in providing relief from depression in these patients [13, 27, 41]**.** Therefore, depression treatment may reduce healthcare expenditures by lowering mental health-related expenditures. Additionally, individuals with depression have been shown to have high utilizations of healthcare services [20, 38], and coexisting depression can worsen other medical conditions by adversely affecting medication adherence [14] and self-care regimens [21]. Thus, healthcare expenditures may be reduced when depression is effectively treated and patients decrease their healthcare utilization, improve their adherence to other chronic disease medications, and administer better self-care regimens.

Our results show that depression treatment modalities are associated with reductions in T2DM-related healthcare expenditures, a finding which is comparable to limited evidence from previous research. For example, Lustman et al. showed that among 51 individuals with T2DM and depression, 10 weeks of cognitive behavioral therapy was significantly associated with reduced glycated hemoglobin (HbA1c) levels, an indicator of lower blood sugar levels and diabetes management, in the intervention group at 6 month after follow-up (intervention vs control: 9.5 % vs 10.9 %; *P* = 0.03). Evidence from randomized controlled trials of collaborative care, which often includes depression treatment with antidepressants as well as psychotherapy (either initiated together or in a stepped care approach based on response to initial treatment), has shown significant reductions in HbA1c levels in the intervention group compared to the control group [3]. These studies support our findings that depression treatment modalities with psychotherapy can reduce T2DM-related expenditures. Our study focused on patients with T2DM and followed them for depression and its treatment. There are debates on the causal pathway of diabetes and depression indicating the possibility that there is reverse causality between diabetes and depression. The current study did not explore such issues.

Across all coexisting condition types, depression treatment with both antidepressants and psychotherapy was associated with reduced total healthcare expenditures. Several studies, including multiple randomized clinical trials [4, 10, 17] and meta-analyses [8, 9] have shown that, among individuals with depression, combined treatment with both pharmacotherapy and psychotherapy significantly reduced depression symptoms and dropout rates. Treatment with both antidepressants and psychotherapy has also been shown to have long term benefits including preventing relapse and increasing depression treatment adherence [31, 45]. Therefore, by improving depression-related outcomes, treatment with both antidepressants and psychotherapy may help to reduce total healthcare expenditures.

Interestingly, unlike our finding that treatment with both antidepressants and psychotherapy was effective at decreasing total healthcare expenditures in each comorbid patient population, other depression treatment categories were not as uniformly associated with reductions in healthcare expenditures. For individuals with either concordant or discordant conditions, antidepressant treatment alone reduced total healthcare expenditures. Depression treatment with only psychotherapy reduced total and T2DM-related healthcare expenditures among those with a high burden of coexisting conditions, such as those with dominant conditions, and also individuals with both concordant and discordant conditions. These results indicate that the choice of treatment needs to be prioritized based on the type of coexisting chronic physical condition and should be considered in addition to the preferred treatment choice of the physician or patient.

The study findings have important implications in the context of new payment models such as “bundled payment”, where providers or facilities are paid a single lump-sum payment for all services in relation to treating a condition or providing a treatment. The results of this study indicate that certain depression treatment types are ineffective in reducing healthcare expenditures. For example, healthcare expenditures were not reduced by depression treatment with only antidepressants in the presence of dominant conditions. Therefore, among individuals with T2DM and coexisting dominant conditions, healthcare systems should not expect economic benefits from initiating treatment for newly-diagnosed depression with antidepressants under bundled payment systems. Our results also have implications for “benchmarking” approaches used by Accountable Care Organizations (ACOs), where expenditure patterns of beneficiaries in the past three years are used to set expenditure “benchmarks” based on risk adjustment models. Our analyses indicate that total healthcare expenditures among Medicaid beneficiaries with T2DM, newly-diagnosed depression, and coexisting conditions may vary both by the coexisting condition type and the method of depression treatment received. Therefore, while setting benchmarks for this patient population, risk adjustments for coexisting condition type and depression treatment modality should be taken into consideration.

There are several strengths of our study, some of which are unprecedented. First, Medicaid claims data spanning multiple years from three states were used in this study, which allowed us to efficiently follow a large cohort of patients for a long period of time across a variety of providers. Second, the study included adults with multiple comorbid conditions, and this patient population is often ignored in clinical trials of depression treatment. Third, the economic consequences of depression treatment were observed in real world settings instead of the controlled environment of clinical trials. The use of a repeated measures design allowed us to study the association between depression treatment and healthcare expenditure over time, instead of aggregating expenditures at the end of follow-up. To the best of the authors’ knowledge, such a design has not yet been adopted by any other study in this area. Fourth, since the association between depression treatments and healthcare expenditures were adjusted for inverse probability treatment weights, selection bias due to differences in observed characteristics in the depression treatment groups could be controlled in the analyses.

However, as a population-based study using Medicaid and administrative claims data, our study shares the limitations often found in observational studies. As administrative claims data can only identify diseases through diagnosis codes, our study could have potentially underestimated newly-diagnosed depression owing to undiagnosed depression and under-coding of depression. Indeed, identifying depression is one of the more difficult problems in administrative data research and perfection may not be attainable. However, the use of diagnosis codes recommended by HEDIS by health plans in order to identify depression claims offers a particularly attractive alternative to the complications associated with prospective surveys using medical record data. Additionally, T2DM and the coexisting chronic physical condition types were also identified using diagnosis codes in medical claims. Incomplete or erroneous records submitted by healthcare providers, limited clinical detail in the ICD-9-CM codes, and inaccurate demographic information might limit the accuracy of administrative data. The duration of T2DM and the physical comorbid chronic conditions were not available and could not be adjusted for in regression analyses. Further, the treatment choices are likely to be partially determined by the severity of the disease in this observational study. Pharmacotherapy may be prescribed in severe cases of depression and the effect of pharmacotherapy on expenditures may partially reflect severity of the illness. Although we controlled for selection bias in observed variables through an IPTW adjustment, a number of unmeasured factors such as patient preferences could influence both treatment choices and treatment outcome. Finally, the study included fee-for-service Medicaid beneficiaries enrolled in three states, and the results might not be fully generalizable to the entire Medicaid population.

## Conclusion

Among working age adults with T2DM, a comorbid chronic physical condition, and newly diagnosed depression, depression treatment can produce cost-savings to Medicaid. Treating depression with antidepressants and psychotherapy combined may be the best method to achieve consistent reductions in expenditures across all types of coexisting chronic physical conditions. For specific modalities of depression treatment (e.g. antidepressant or psychotherapy administered singly), cost-reductions will depend on the coexisting chronic physical condition type.

## Abbreviations

ACOs, Accountable Care Organizations; AHRF, area health resource files; CMHC, Community mental health clinic; CPI, consumer price index; CPT, current procedural terminology; FQHC, Federally qualified health clinic; HPSA, Health Professional Shortage area; ICD-9-CM, International Classification of Diseases 9th revision; IL, Illinois; IPTW, Inverse Probability of Treatment Weighting; MAX, medicaid analytic extract; NY, New York; T2DM, type 2 diabetes mellitus; TX, Texas

## Appendix

**Table 4:** ICD-9-CM codes for identifying coexisting chronic physical condition type. We provide the list of ICD-9-CM codes for identifying coexisting chronic physical condition type

Conditions		ICD-9-CM codes
Concordant Conditions		
	Coronary Artery Disease	410,4100,4101,4102,4103,4104,4105,4106,4107,4108,4109,411,4110,4111,4118,41181,41189,412,413,4130,4131,4139,414,4140,41400,41401,41402,41403,41404,41405, 4141,41410,41411, 41419,4148,4149
	Congestive Heart Failure	40201,40211,40291,40401,40411,40491,428,4280,4281,4289
	Arrhythmia	423,4230,4231,4232,4238,4239,42731
	Stroke	431,43301,43311,43321,43331,43381,43391,43401,43411,43491,435,4350,4351,4352,4353,4358,4359,438,4380,4381,43811,43812,4382,4383,4384,4385,43850,4385,43852,43853,4388,43881, 43882,43889,4389
	Peripheral Vascular Disease	2507,4402,44020,44021,44022,44023,44024,44029,4408,4409,4422,4423,443,4430, 4431,4438,44381, 44389,4439,44422,44481
	Peripheral Vascular Disease-gangrene	7854
	Renal	40311,40391,40412,40413,40492,40493, 585,586,587, 2741,27410,27411,27419,40310,40390,40410,40411,40490,40491, 581,5810,5811,5812,5813,5818,5819, 582,5820,5821,5822,5824,5828,58281,58289,5829,583,5830,5831,5832,5834,5836, 5837, 5838,58381,58389,5839, 5900,59000,59001,5936,5939, 75312,75313,75314
	Chronic Renal Failure	
	Chronic Pathophysiology	
	Diabetic Nephropathy	2504,25040,25041,25042,25043
	Acute Renal Failure and Disease	40300,40301,40400,40401,40402,40403,40501,4533,584,5845,5846,5847,5848,5849,580,5800,5804,5808,58081,58089,5809,5901,59010,59011,5902,5903,5908,59080, 59081,59381,866,8660,86600,86601,86602,86603,8661,86610,86611,86612,86613
	Retinopathy (excludes advanced retinopathy, blindness)	3620,36201,25050,25051,25052,25053
	Ulcer	700,68110,68111,6827,7071,73076,73077
	Other Diabetes Related Complications	25002,25003,25010,25011,25012,25013,25020,25021,25022,25023,25030,25031, 25032,25033
	Uncontrolled Diabetes	
	Short Term Diabetes	
Discordant Conditions		
	Gastro Intestinal Tract Related Disorders:	5301,5302,5303,53081,531,532,533,534,555,556,56211,56213,574, 575,576,070
	GERD/Esophagitis	
	Peptic ulcers	
	Inflammatory Bowel Disease	
	Diverticulitis	
	Gall bladder disease and stone	
	Viral hepatitis	
	Chronic Obstructive Pulmonary Disease	490,491,492,493,495,496,500,501,502,503,504,505,5064
	Gout	274,712
	Hip problems	71905,71915,71925,71935,71945,71955,71965,71975,71985,71995,7265,73314,73315,73342,820
	Lower back pain	720,7213,72142,72210,72252,72273,72283, 72293,72402,7242,7243,7244,7245,7246,7247,7248,7249
	Osteoarthritis	715
	Other arthritis	716
	Rheumatoid arthritis	714
	Connective tissue rheumatological disease	7100,7101,7104,725
	Blindness Single Eye	3696,3697,3698,3699
Dominant Conditions		
	End Stage Renal Disease	E8791,V51,V56,V560,V5631,V5632,V568
	End Stage Liver Disease	5722,5723,5724,5728,4560,4561, 4562,45620,45621, 571
	Blindness Both Eyes/ Advanced Retinopathy	36202,3690,3691,3692,3693,3694
	Cancer	140,141,142,143,144,145,146,147,148,149,150,151,152,153,154,155,156,157,158,159,160,161,162,163,164,165,166,167,168,169,170,171,172,174,175,176,177,178,179,180,181,182,183,184,186,187,188,189,190,191,192,193,194,195,196,197,198,199,200,201,202,203,204,205,206,207,208
	Pre-dementia Cognitive Impairment	294,2941, 29283, 2949,33183,78093,438,3330,3334,3315
	Dementia and Related Conditions	2900,29010,29011,29012,29013,2902,29021,2903,29040,29041,29042,29043,2912, 29410,29411,2948,3310,3311,3312,3317,33182,33189,3319,3320,0461,0463,0941, 29282,3109
	Multiple Sclerosis	340
	Hemiplegia Hemiparesis and Paraplegia	342,3441
	Parkinson’s Disease	332
	Muscular dystrophy	359
	Spinal cord injury	80600,80601,80602,80603,80604,80605,80606,80607,80608,80609,8061, 9520, 34400,34401,34402,34403,34404,34409
	Epilepsy	345
	Gastropareis	5363
	AIDS	042
